# Integrative Transcriptomic Analysis Identifies Shared Immune–Fibrotic Transcriptional Programs Across Crohn’s Disease and Idiopathic Pulmonary Fibrosis

**DOI:** 10.3390/ijms27104428

**Published:** 2026-05-15

**Authors:** Renwei Luo, Qiong Zhang, Qinglu Fan, Qingyun Chen, Zhihao Nie, Lingxuan Dan, Fengling Luo, Yige Cao, Songping Xie

**Affiliations:** 1 Department of Thoracic Surgery, Renmin Hospital of Wuhan University, Wuhan 430060, China; 2023203020029@whu.edu.cn (R.L.); rm003718@whu.edu.cn (Q.Z.);; 2 State Key Laboratory of Virology, Hubei Province Key Laboratory of Allergy and Immunology, Department of Immunology, Taikang Medical School (School of Basic Medical Sciences), Wuhan University, Wuhan 430060, China

**Keywords:** Crohn’s disease, idiopathic pulmonary fibrosis, integrative transcriptomic analysis, immune–fibrotic transcriptional programs, ZNF395, EEF2K, BAHD1

## Abstract

Idiopathic pulmonary fibrosis (IPF) and Crohn’s disease (CD) share overlapping immune and fibrotic processes, yet their convergent molecular mechanisms remain poorly defined. Here, we performed an integrative transcriptomic analysis of nine public datasets to identify shared transcriptional signatures across IPF and CD. The main discovery and validation analyses were based on bulk transcriptomic datasets and combined differential expression profiling, weighted gene co-expression network analysis, and machine-learning–based feature prioritization. We identified 28 shared disease-associated module genes, from which three core genes—ZNF395, EEF2K, and BAHD1—were prioritized based on reproducibility and biological consistency. Functional enrichment analysis revealed their involvement in immune regulation, protein homeostasis, and stress-response pathways. Immune deconvolution and supportive single-cell RNA-sequencing further suggested associations between these genes and T-cell and myeloid cell populations, suggesting coordinated immune-fibrotic regulation. Experimental validation in a repetitive bleomycin challenge model and TGF-β1-stimulated fibroblasts showed consistent downregulation of these genes during fibrotic remodeling, supporting their association with fibrosis-related transcriptional states. Collectively, our study identifies conserved immune–fibrotic transcriptional programs shared across intestinal inflammation and pulmonary fibrosis, providing a hypothesis-generating molecular framework for understanding extraintestinal pulmonary involvement in Crohn’s disease and prioritizing candidate genes for future mechanistic investigation.

## 1. Introduction

Idiopathic pulmonary fibrosis (IPF) is a progressive interstitial lung disease characterized by aberrant tissue remodeling, excessive extracellular matrix deposition, and irreversible decline in lung function [1,2]. Its global incidence continues to rise, partly owing to improved diagnostic recognition [3,4]. Although established risk factors—including smoking, genetic susceptibility, and environmental exposures—contribute to disease onset, the molecular mechanisms driving IPF pathogenesis remain incompletely understood [5].

Inflammatory bowel disease (IBD), encompassing Crohn’s disease (CD) and ulcerative colitis (UC), is a chronic relapsing inflammatory disorder of the gastrointestinal tract. Extraintestinal manifestations (EIMs) occur in up to 50% of IBD patients [6,7], among which pulmonary involvement is clinically significant yet frequently underrecognized. Reported respiratory complications include chronic bronchitis, pulmonary vasculitis, eosinophilic pneumonia, granulomatous lung disease, and IPF [8,9,10]. Epidemiological studies suggest that patients with CD may have a higher risk of developing IPF than patients with UC [11]. This observation raises the possibility of a closer pathogenic relationship between CD and pulmonary fibrosis.

Mechanistic parallels also support this association. Both CD and IPF involve dysregulated immune responses, fibroblast activation, and abnormal tissue repair. Shared profibrotic pathways, including transforming growth factor-β (TGF-β) signaling and NOD-like receptor–related inflammatory cascades, may contribute to fibrogenesis in both intestinal and pulmonary tissues [12,13,14,15]. In addition, ectopic expression of intestinal chemokines and adhesion molecules, such as CCL25 and mucosal addressin cell adhesion molecule-1 (MAdCAM-1), has been reported in extraintestinal inflammatory settings [16,17]. These signals may promote aberrant lymphocyte trafficking to distant organs and could contribute to pulmonary manifestations in CD.

Despite these epidemiological and mechanistic observations, critical gaps remain. Specifically, the transcriptional programs that may be shared between CD and IPF—and that could underlie their clinical and immunopathological association—have not been systematically characterized. Moreover, the absence of well-defined cohorts of CD patients with concomitant IPF has limited direct molecular investigation of this disease intersection.

To address this gap, we used an integrative transcriptomic strategy to identify shared molecular signatures across CD and IPF. The main discovery and validation analyses were based on bulk transcriptomic datasets, combined with co-expression network analysis, machine-learning–based feature prioritization, and immune infiltration profiling. In addition, single-cell RNA sequencing was used as a separate supportive analysis to provide cellular context for the identified core genes. We further performed experimental validation in murine and cellular fibrosis-associated models to assess the biological relevance of the prioritized genes. This study was designed to generate mechanistic hypotheses about molecular convergence between intestinal inflammation and pulmonary fibrosis, rather than to define disease-specific biomarkers for confirmed CD-associated IPF.

## 2. Results

### 2.1. Identification of Shared Transcriptional Alterations Between IPF and CD

**Figure 1:**
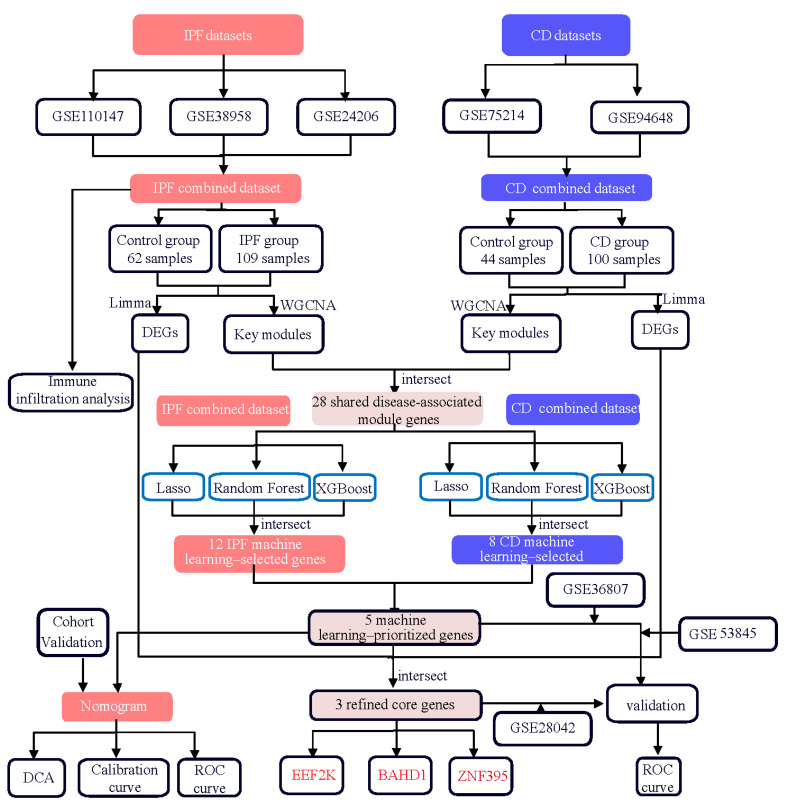
Overview of the integrative transcriptomic analysis workflow. Bulk transcriptomic datasets from lung tissues of idiopathic pulmonary fibrosis (IPF) and colonic tissues of Crohn’s disease (CD) were analyzed independently. Differential expression analysis and weighted gene co-expression network analysis (WGCNA) were performed within each disease to identify disease-associated gene sets. Shared candidate genes were defined by gene-level intersection between IPF and CD. Machine learning–based feature prioritization (LASSO, random forest, and XGBoost) was applied to refine shared gene signatures. The resulting gene sets were evaluated using ROC analysis, calibration curves, and decision curve analysis, and further assessed in independent validation cohorts. Immune cell infiltration analysis was conducted to explore immune-related transcriptional features associated with the shared signatures.

As illustrated in Figure 1, this study followed a stepwise integrative analytical workflow encompassing data preprocessing and batch correction, differential expression analysis, co-expression network construction, machine learning–based feature selection, and multi-cohort validation. Briefly, transcriptomic datasets from IPF lung tissues and CD intestinal tissues were first independently processed and then integrated to identify shared molecular signatures across pulmonary and intestinal disease contexts.

After data preprocessing and batch correction, integrated transcriptomic profiles were obtained for both diseases, comprising 109 IPF patients and 62 controls, as well as 100 CD patients and 44 controls. Batch correction effectively harmonized transcriptional patterns across datasets, as demonstrated by principal component analysis (Figures S1 and S2).

**Figure 2:**
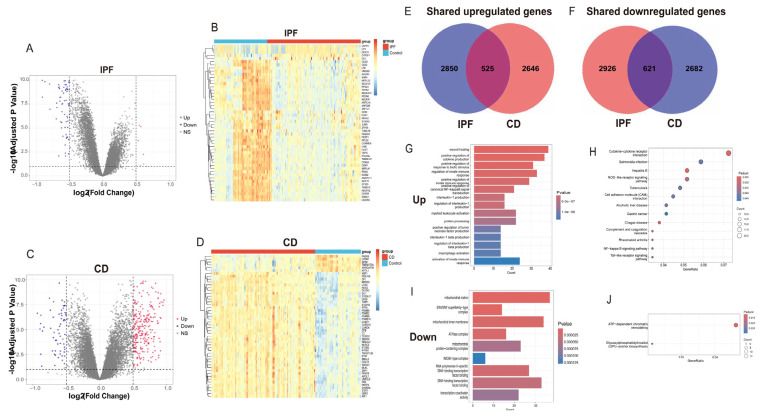
Identification of shared differentially expressed genes between IPF and CD. (**A**–**D**) Volcano plots illustrating DEGs identified in the integrated IPF and CD cohorts, respectively. (**E**,**F**) Venn diagrams showing the overlap of upregulated (**E**) and downregulated (**F**) DEGs between IPF and CD. (**G**,**H**) Gene Ontology (GO) biological process enrichment (**G**) and KEGG pathway enrichment (**H**) analyses of commonly upregulated DEGs, highlighting immune- and inflammation-related pathways. (**I**,**J**) GO (**I**) and KEGG (**J**) enrichment analyses of commonly downregulated DEGs. Enrichment results are shown based on adjusted *p* values < 0.05.

Comparative transcriptomic analysis revealed a substantial overlap in differentially expressed genes (DEGs) between IPF and CD (Figure 2A–D), indicating shared molecular alterations across distinct tissue environments. In total, 525 commonly upregulated and 621 commonly downregulated shared DEGs were identified (Figure 2E,F).

To improve biological interpretability, functional enrichment analyses were conducted separately for the shared upregulated and downregulated DEGs (Figure 2G–J). Gene Ontology (GO) enrichment analysis of upregulated genes demonstrated predominant involvement in immune- and inflammation-related biological processes, including positive regulation of cytokine production, regulation of interleukin-1 production, innate immune activation, and responses to inflammatory and biotic stimuli (Figure 2G). Consistently, KEGG pathway analysis highlighted enrichment in immune-associated signaling pathways such as cytokine–cytokine receptor interaction, Toll-like receptor signaling, NOD-like receptor signaling, NF-κB signaling, and complement and coagulation cascades (Figure 2H).

In contrast, the shared downregulated DEGs were mainly enriched in GO terms related to cellular organization and transcriptional regulation, including mitochondrial components, protein-containing complexes, and DNA-binding transcription factor activity (Figure 2I). Correspondingly, KEGG pathway analysis revealed enrichment in ATP-dependent chromatin remodeling and glycosylphosphatidylinositol (GPI)-anchor biosynthesis pathways (Figure 2J). Collectively, these analyses showed concurrent enrichment of immune-inflammatory pathways among upregulated genes and regulatory or structural cellular processes among downregulated genes.

To clarify the analytical hierarchy used in this study, the overlapping differentially expressed genes identified at this stage were defined as shared DEGs, representing broad transcriptional commonalities between IPF and CD. We next integrated weighted gene co-expression network analysis to identify shared disease-associated module genes, reflecting gene sets most strongly linked to disease status in both conditions. From these candidates, machine learning algorithms were applied to derive machine learning–prioritized genes, which were subsequently refined by intersecting them with the shared DEG set to generate a final set of refined core genes for downstream interpretation and experimental validation.

### 2.2. Identification of Disease-Associated Co-Expression Modules and Shared Gene Signatures

**Figure 3:**
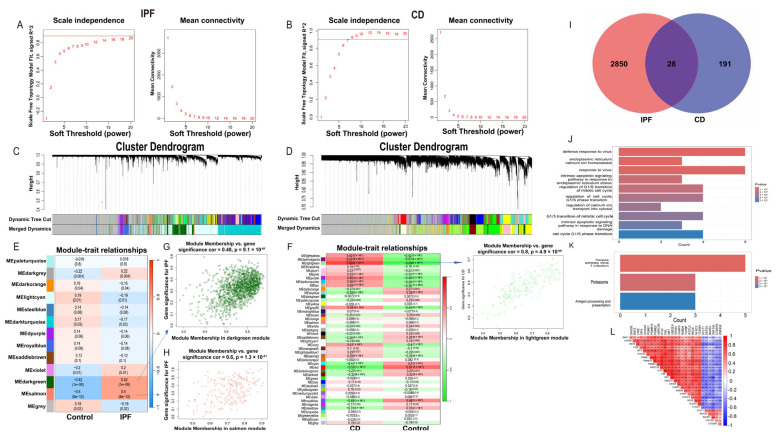
WGCNA identifies disease-associated co-expression modules and shared gene signatures. (**A**,**B**) Scale-free topology fitting curves used to determine optimal soft-thresholding powers for the integrated IPF (β = 18, A) and CD (β = 7, B) datasets. (**C**,**D**) Gene dendrograms and module assignments identified in the IPF (**C**) and CD (**D**) cohorts. (**E**,**F**) Heatmaps depicting correlations between module eigengenes and disease status in IPF (**E**) and CD (**F**). (**G**,**H**) Scatter plots showing relationships between module membership (MM) and gene significance (GS) for IPF-associated salmon (**G**) and darkgreen (**H**) modules. (**I**) Venn diagram illustrating the intersection of IPF-associated modules (salmon and darkgreen) and the CD-associated lightgreen module, yielding 28 shared genes. (**J**,**K**) GO (**J**) and KEGG (**K**) enrichment analyses of the 28 shared genes. (**L**) Correlation heatmap showing pairwise transcriptional relationships among the 28 shared genes.

Consistent with the analytical framework outlined in Figure 1, weighted gene co-expression network analysis (WGCNA) was subsequently applied to the integrated datasets to identify disease-associated gene modules. WGCNA identified 13 distinct co-expression modules in the integrated IPF dataset and 45 modules in the combined CD cohort (Figure 3C,D).

Correlation analysis between module eigengenes and disease status revealed that, in the IPF cohort, the salmon (r = 0.50, *p* = 6 × 10^−12^) and darkgreen (r = 0.42, *p* = 1 × 10^−8^) modules exhibited the strongest positive associations with disease status (Figure 3E). Both modules demonstrated robust module membership–gene significance (MM–GS) correlations (salmon: r = 0.60, *p* = 1.3 × 10^−21^; darkgreen: r = 0.48, *p* = 9.1 × 10^−87^) (Figure 3G,H), supporting their biological relevance to IPF pathogenesis. Together, these two modules comprised 1698 IPF-associated genes.

In the CD cohort, the lightgreen module showed the strongest correlation with disease status (r = 0.69, *p* = 2 × 10^−21^) and exhibited coherent MM–GS relationships (Figure 3D,F). Based on its robust disease association, this module was selected for cross-disease comparison.

Intersection of genes derived from the IPF-associated salmon and darkgreen modules with those from the CD-associated lightgreen module yielded 28 shared disease-associated module genes (Figure 3I), representing convergent transcriptional programs most strongly linked to disease in both conditions. Because the objective of this study was to identify shared disease-associated candidates across CD and IPF rather than classical intramodular hub genes within a single disease network, genes from the selected disease-associated modules were carried forward for cross-disease intersection analysis and subsequent machine-learning–based prioritization.

Functional enrichment analysis of these 28 shared disease-associated module genes revealed a focused biological profile centered on cellular stress responses and proteostasis (Figure 3J,K). GO enrichment analysis demonstrated significant involvement in endoplasmic reticulum (ER) calcium ion homeostasis, intrinsic apoptotic signaling in response to ER stress and DNA damage, as well as G1/S transition of the mitotic cell cycle (Figure 3J). Consistently, KEGG pathway analysis highlighted enrichment in antigen processing and presentation and the proteasome pathway (Figure 3K), indicating coordinated regulation of protein turnover and immune surveillance.

Correlation analysis further revealed structured co-expression relationships among these genes, including a strong positive correlation between PML and IFI35 (r = 0.84, *p* < 0.0001) and a strong negative correlation between ZNF395 and IRF1 (r = −0.64, *p* < 0.0001) (Figure 3L).

### 2.3. Machine Learning–Based Prioritization of Shared Disease-Associated Gene Signatures Across CD and IPF

**Figure 4:**
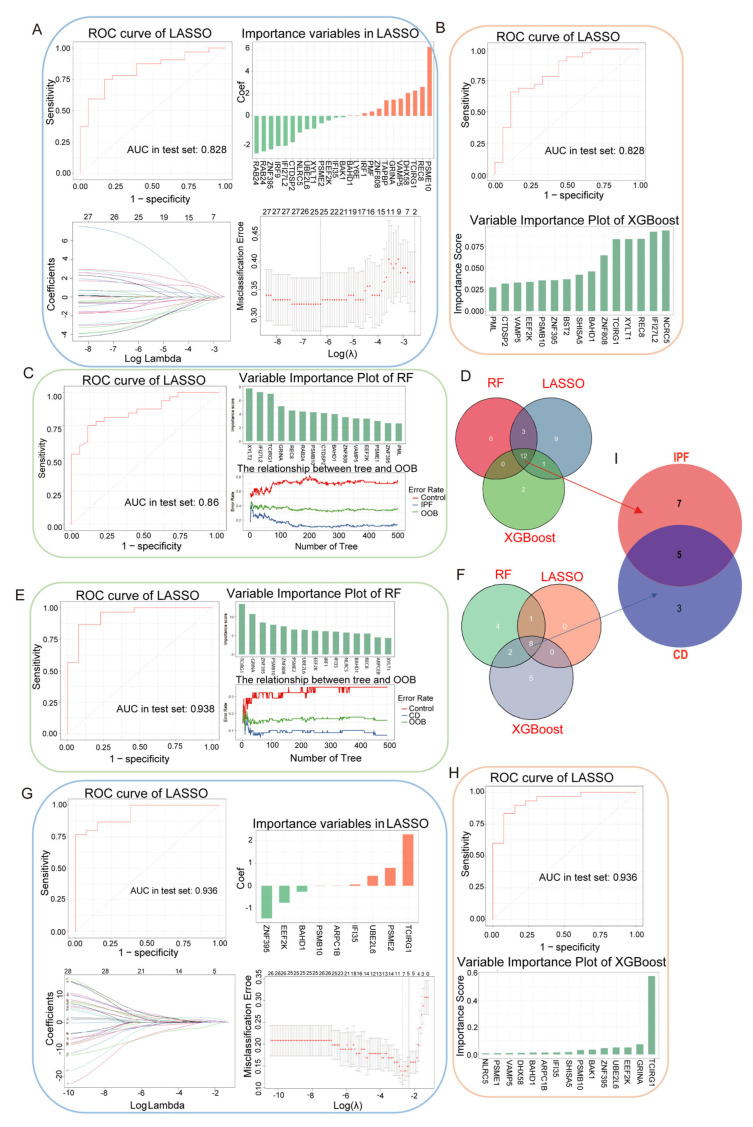
Machine learning–based prioritization of shared disease-associated gene signatures across CD and IPF. (**A**) Feature selection results obtained using LASSO regression in the IPF cohort. (**B**) Feature importance rankings generated by XGBoost analysis in the IPF cohort. (**C**) Variable importance rankings derived from random forest (RF) analysis in the IPF cohort. (**D**) Venn diagram showing the 12 genes consistently prioritized across LASSO, XGBoost, and RF in the IPF cohort. (**E**) Variable importance rankings derived from random forest (RF) analysis in the CD cohort. (**F**) Feature selection results obtained using LASSO regression in the CD cohort. (**G**) Feature importance rankings generated by XGBoost analysis in the CD cohort. (**H**) Venn diagram of the 8 intersecting genes identified across the LASSO, XGBoost, and RF algorithms in the CD cohort. (**I**) Venn diagram of the 5 shared gene signatures between IPF cohort and CD cohort. Using the 28 shared disease-associated module genes as the common candidate set, feature selection was performed separately in the IPF and CD cohorts with LASSO, XGBoost, and RF. Genes consistently prioritized across the three algorithms within each disease context were then compared across the two disease cohorts, yielding five shared machine-learning–prioritized genes (TCIRG1, ZNF395, PSMB10, EEF2K, and BAHD1).

To further refine the 28 shared disease-associated module genes identified from the cross-disease module intersection analysis, a multi-algorithm machine learning strategy was applied as an additional feature prioritization framework. Using these 28 genes as the common candidate set, least absolute shrinkage and selection operator (LASSO) regression, random forest (RF), and extreme gradient boosting (XGBoost) were performed separately in the IPF and CD cohorts. Within each disease context, genes consistently prioritized across the three algorithms were retained, and the overlapping genes identified in both disease cohorts were defined as machine-learning–prioritized genes. This procedure yielded five genes—TCIRG1, ZNF395, PSMB10, EEF2K, and BAHD1—representing a stable set of shared disease-associated features (Figure 4A–I).

**Figure 5:**
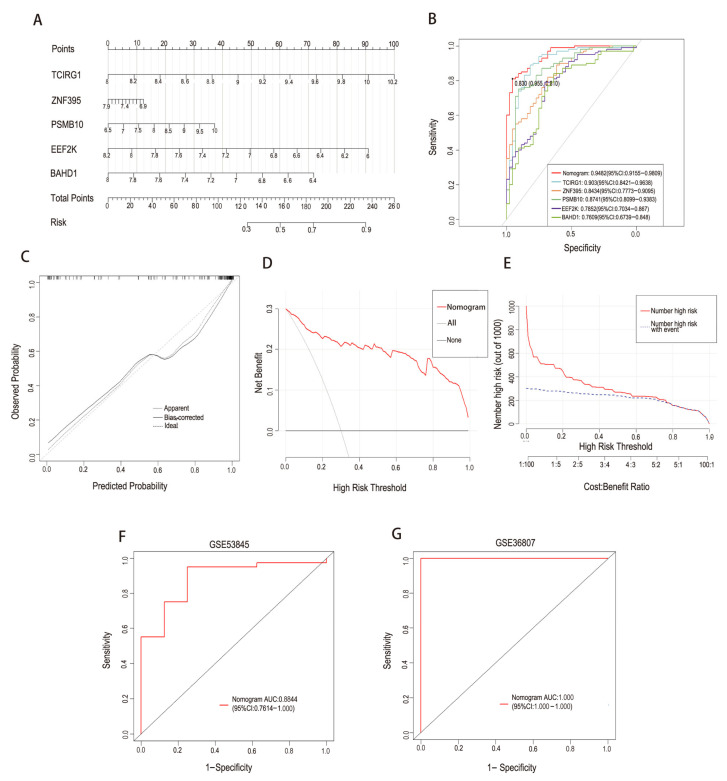
Construction and validation of a five-gene integrative nomogram. (**A**) Nomogram integrating the five machine learning–prioritized genes to summarize their relative contributions to within-disease transcriptional separation. (**B**) Receiver operating characteristic (ROC) curve for the training cohort. (**C**) Calibration curve evaluating agreement between predicted and observed probabilities. (**D**) Decision curve analysis (DCA) illustrating net benefit across a range of threshold probabilities. (**E**) CIC assessment of the nomogram’s predictive utility. (**F**,**G**) ROC curves validating the five-gene signature in independent IPF (GSE53845) and CD (GSE36807) cohorts, respectively. These analyses assess reproducibility within each disease context rather than cross-disease prediction.

To facilitate visualization of the combined discriminatory capacity of these five genes, a logistic regression–based model was constructed and presented in the form of a nomogram (Figure 5A). Notably, this modeling step was intended to summarize and evaluate feature stability and within-disease separation rather than to establish a clinically deployable diagnostic tool for CD-associated pulmonary fibrosis.

The five-gene signature demonstrated stable within-disease discriminatory performance in the training cohort, with an area under the receiver operating characteristic curve (AUC) of 0.948 (Figure 5B). Calibration analysis showed agreement between predicted and observed probabilities (Figure 5C), and decision curve analysis showed separation of model curves across a range of threshold probabilities within disease-specific contexts (Figure 5D). The robustness of this signature was further supported by validation in two independent cohorts—GSE53845 for IPF and GSE36807 for CD—where AUC values of 0.884 and 1.000, respectively, were observed (Figure 5F,G). Importantly, these analyses assess the reproducibility of transcriptional separation within each disease and do not imply cross-disease prediction or clinical risk stratification of IPF in CD patients.

Given the inherent biological differences between peripheral blood and solid organ tissues, validation in peripheral blood mononuclear cells (PBMCs; GSE28042) was performed solely to explore systemic immune relevance and cross-tissue transcriptional consistency, rather than to model pulmonary fibrotic pathology or infer disease prediction.

**Figure 6:**
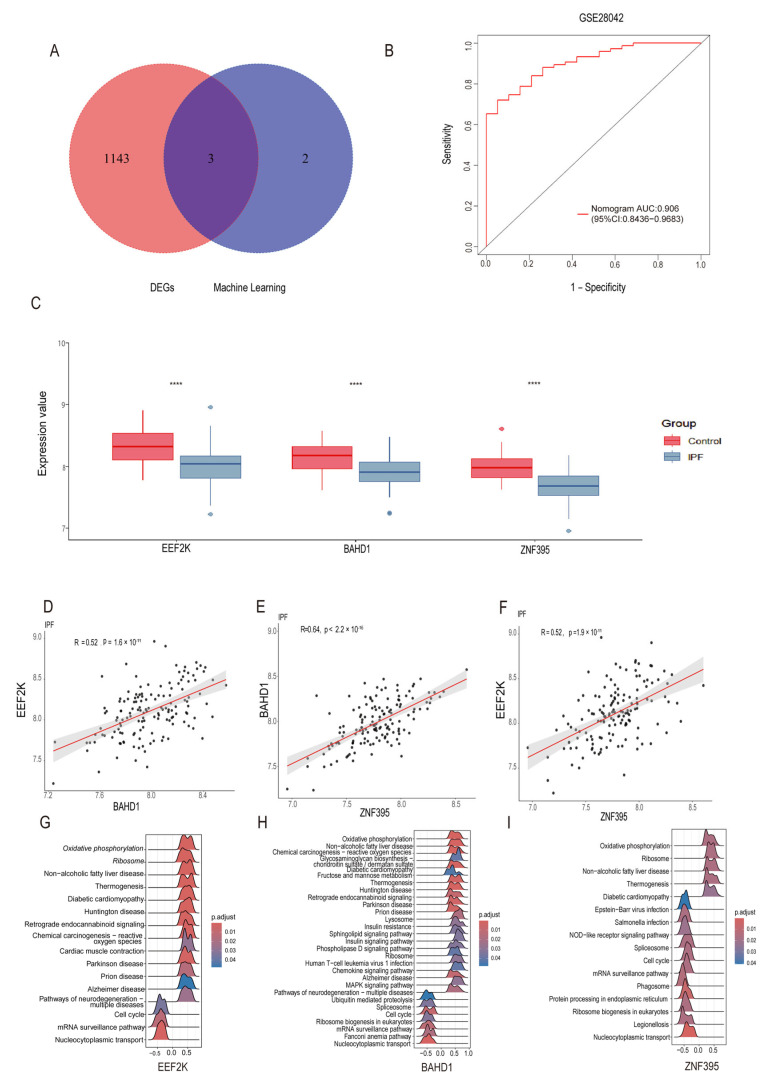
Refinement to a three-gene core signature and functional characterization of the refined core genes. (**A**) Venn diagram showing the intersection of machine learning–prioritized genes with shared DEGs, yielding ZNF395, EEF2K, and BAHD1. (**B**,**C**) ROC curve evaluating the three-gene signature in a PBMC-based exploratory IPF cohort (GSE28042), used to assess systemic immune relevance and cross-tissue transcriptional consistency rather than to directly validate lung fibrotic pathology. (**D**–**F**) Pairwise correlation analyses among ZNF395, EEF2K, and BAHD1 in the same PBMC-based exploratory cohort. (**G**–**I**) Single-gene–based GSEA for EEF2K (**G**), BAHD1 (**H**), and ZNF395 (**I**), showing convergence on pathways related to ribosome function, oxidative phosphorylation, mRNA surveillance, and cellular stress responses. **** *p* < 0.0001.

While the five-gene set demonstrated strong and reproducible discriminatory capacity, we next sought to derive a more parsimonious and biologically interpretable core gene signature. To this end, the five machine learning–derived genes were intersected with the overlapping DEGs identified in the initial comparative analysis, thereby integrating statistical robustness with biological consistency. This refinement step yielded three final core genes—ZNF395, EEF2K, and BAHD1 (Figure 6A).

As a blood-based exploratory validation aimed at assessing systemic immune relevance and cross-tissue transcriptional consistency, the discriminatory capacity of this three-gene signature was further examined in an independent PBMC-based IPF cohort (GSE28042), where it achieved an AUC of 0.906 (Figure 6B). These findings support the robustness of the refined signature across tissue contexts, but are not intended to directly validate lung fibrotic pathology.

To further assess whether the refined signature retained coordinated transcriptional behavior across tissue contexts, pairwise correlation analyses were performed in the same PBMC-based exploratory cohort (GSE28042). All three genes—ZNF395, EEF2K, and BAHD1—exhibited significant positive correlations at the transcriptional level, supporting cross-tissue consistency of the shared signature rather than directly validating lung fibrosis biology.

Among these associations, ZNF395 showed the strongest correlation with BAHD1 (Pearson’s r = 0.64, *p* < 0.001; Figure 6D), while EEF2K was moderately but consistently correlated with both ZNF395 (r = 0.52, *p* < 0.001; Figure 6E) and BAHD1 (r = 0.52, *p* < 0.001; Figure 6C). Stable positive correlations were observed across all gene pairs, consistent with coordinated expression of the three genes across disease-relevant tissue contexts.

To provide biological context for the three core genes, single-gene–based gene set enrichment analysis (GSEA) was performed for ZNF395, EEF2K, and BAHD1 (Figure 6F–H). Across the three genes, consistently enriched pathways included those involved in ribosome function and biogenesis, oxidative phosphorylation, thermogenesis, mRNA surveillance, and nucleocytoplasmic transport. In addition, ZNF395 and BAHD1 showed enrichment in pathways associated with cell cycle regulation, whereas EEF2K demonstrated prominent associations with translational control and metabolic pathways.

Several enriched pathways were also related to stress-responsive and degeneration-associated processes. Collectively, these enrichment patterns showed convergence on pathways related to ribosome function, oxidative phosphorylation, mRNA surveillance, and cellular stress responses.

### 2.4. Core Genes Are Associated with Immune Cell Infiltration in IPF

**Figure 7:**
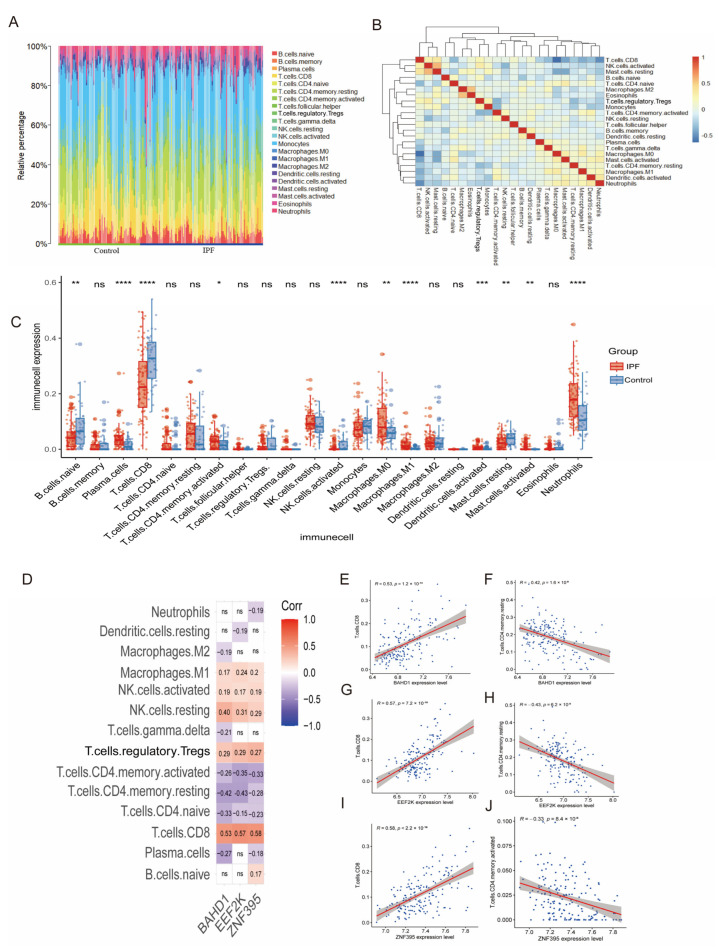
Immune infiltration analysis and associations with core gene expression in IPF. (**A**) Relative proportions of 22 immune cell subsets estimated using CIBERSORT. (**B**) Correlation matrix depicting interactions among immune cell populations. (**C**) Comparative analysis of immune cell proportions between IPF patients and controls. (**D**) Heatmap summarizing correlations between core gene expression and immune cell subsets. (**E**–**J**) Scatter plots illustrating significant associations between ZNF395, EEF2K, BAHD1 expression and CD8^+^ and CD4^+^ memory T-cell populations. Correlations are descriptive and do not imply causality. *IPF:* Idiopathic pulmonary fibrosis. * *p* < 0.05; ** *p* < 0.01; *** *p* < 0.001; **** *p* < 0.0001; ns, not significant.

To characterize immune cell infiltration patterns in IPF, the relative abundances of 22 immune cell subsets were estimated using the CIBERSORT algorithm. The immune cell composition of each sample is summarized in Figure 7A. Intercellular correlation analysis revealed structured immune interactions, with the strongest positive correlation observed between resting mast cells and activated NK cells (r = 0.57, *p* < 0.05), and the strongest negative correlation between CD8^+^ T cells and M0 macrophages (r = −0.64, *p* < 0.05) (Figure 7B).

Comparative analysis between IPF patients and controls demonstrated significant alterations in immune cell composition (Figure 7C). IPF samples exhibited increased proportions of CD8^+^ T cells, naïve B cells, activated NK cells, and resting mast cells, whereas reduced proportions of plasma cells, activated CD4^+^ memory T cells, M0 and M1 macrophages, activated dendritic cells, activated mast cells, and neutrophils were observed.

To explore the immunological relevance of the identified core genes, correlations between gene expression levels and immune cell proportions were analyzed (Figure 7D). EEF2K and BAHD1 expression showed significant positive correlations with CD8^+^ T cells (EEF2K: r = 0.53; BAHD1: r = 0.57; both *p* < 0.05) and negative correlations with resting CD4^+^ memory T cells (EEF2K: r = −0.42; BAHD1: r = −0.43; both *p* < 0.05) (Figure 7E–H). Similarly, ZNF395 expression was positively correlated with CD8^+^ T cells (r = 0.58, *p* < 0.05) (Figure 7I) and negatively correlated with activated CD4^+^ memory T cells (r = −0.33, *p* < 0.05) (Figure 7J).

Taken together, these results showed significant associations between ZNF395, EEF2K, and BAHD1 expression and immune cell composition in IPF, particularly within cytotoxic T-cell– and T-cell memory–related compartments.

### 2.5. Experimental Validation of the Core Genes in In Vivo and In Vitro Fibrosis-Associated Models

**Figure 8:**
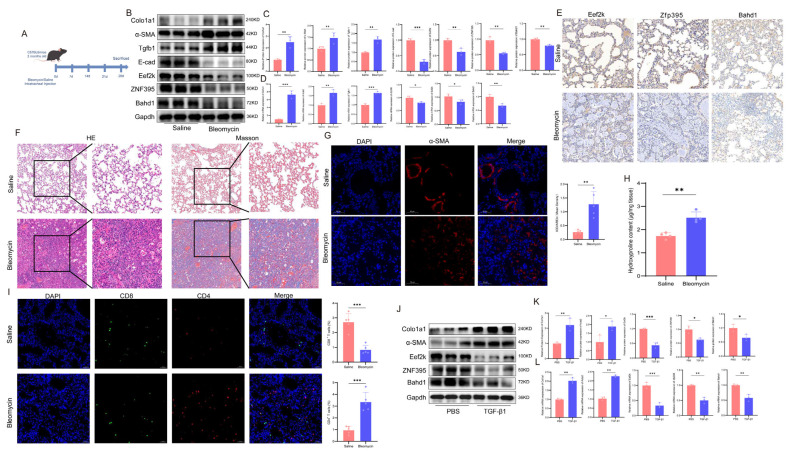
Experimental Validation of the Core Genes in In Vivo and In Vitro Fibrosis-Associated Models. (**A**) Schematic timeline of the repetitive intratracheal bleomycin challenge protocol and tissue collection. (**B**,**C**) Representative Western blot images and densitometric quantification of Eef2k, Bahd1, Zfp395, and fibrosis-associated markers (Col1a1, α-SMA, Tgfb1, and E-cadherin) in lung tissues. (**D**) qPCR validation of Col1a1, Acta2, Tgfb1, zfp395, Eef2k and Bahd1. (**E**) Immunohistochemistry of core genes (Eef2k, Bahd1, and Zfp395) in lung tissues from mice subjected to repetitive bleomycin challenge and saline-treated controls on day 28; scale bar = 50 μm. (**F**) Representative H&E and Masson’s trichrome staining of lung tissues. The right panels show higher-magnification views of the corresponding regions in the left panels; scale bar = 50 μm. (**G**) Immunofluorescence staining of α-SMA in lung tissues; scale bar = 50 μm. (**H**) Hydroxyproline content in lung tissues. (**I**) Immunofluorescence staining of CD4^+^ and CD8^+^ T cells in lung tissues; scale bar = 50 μm; Representative immunofluorescence staining of lung tissues showing DAPI (blue), CD8 (green), and CD4 (red) in the Saline and Bleomycin groups (**J**,**K**) Representative Western blot images and densitometric quantification of Eef2k, Bahd1, Zfp395, and fibrosis-associated markers (Col1a1 and α-SMA) in TGF-β1-stimulated L929 cells. (**L**) qRT-PCR analysis of Col1a1, Acta2, Zfp395, Eef2k, and Bahd1 expression in L929 cells. Overall, these findings show that Zfp395, Eef2k, and Bahd1 are consistently downregulated in repetitive bleomycin-associated lung remodeling and in TGF-β1-stimulated fibroblasts, supporting their association with fibrosis-related expression changes rather than demonstrating a direct antifibrotic function. Data are presented as mean ± SD. * *p* < 0.05, ** *p* < 0.01, *** *p* < 0.001. Statistical significance was determined by Student’s *t*-test.

To experimentally validate the expression patterns of the core genes identified by integrative bioinformatics analyses, we performed both in vivo and in vitro assays using canonical pulmonary fibrosis-associated models (Figure 8). In the repetitive bleomycin-induced lung remodeling model, histopathological analyses using hematoxylin–eosin (HE) and Masson’s trichrome staining revealed marked alveolar architectural disruption and extensive collagen deposition compared with saline-treated controls (Figure 8F). Consistently, hydroxyproline assays demonstrated a significant increase in total collagen content in bleomycin-treated lung tissues (Figure 8H), confirming successful induction of fibrotic remodeling. Western blotting and quantitative PCR further showed significant upregulation of established fibrotic markers, including Col1a1, α-SMA (Acta2) and Tgfb1, following bleomycin challenge (Figure 8B–D).

In parallel, the expression levels of the three core genes—Eef2k, Zfp395, and Bahd1—were markedly reduced in fibrotic lung tissues. Both protein and mRNA analyses demonstrated significant downregulation of these genes in bleomycin-treated mice compared with controls (Figure 8B–E). Immunohistochemical staining further confirmed decreased expression of Eef2k, Zfp395, and Bahd1 within fibrotic regions of lung tissue (Figure 8E), supporting the transcriptomic findings derived from human datasets. Immunofluorescence analysis additionally revealed altered T-cell distributions in fibrotic lungs, characterized by increased CD4^+^ T-cell signals and reduced CD8^+^ T-cell signals following bleomycin treatment (Figure 8I). These observations suggest that fibrotic remodeling is accompanied by altered T-cell–associated immune landscapes, although the direction of specific T-cell subset changes may vary across species, tissues, analytical methods, and disease stages.

To further assess the relevance of these biomarkers in a cellular fibrosis model, L929 fibroblasts were stimulated with TGF-β1 in vitro. TGF-β1 treatment induced robust upregulation of fibrotic markers Col1a1 and α-SMA at both the mRNA and protein levels (Figure 8J–L). Notably, Eef2k, Zfp395, and Bahd1 expression was consistently suppressed following TGF-β1 stimulation, mirroring the expression trends observed in vivo.

Collectively, these in vivo and in vitro findings showed that Eef2k, Zfp395, and Bahd1 were consistently downregulated during fibrotic remodeling and were inversely associated with canonical fibrotic activation.

### 2.6. Single-Cell Transcriptomic Analysis Reveals Cell-Type-Specific Expression of the Core Genes

**Figure 9:**
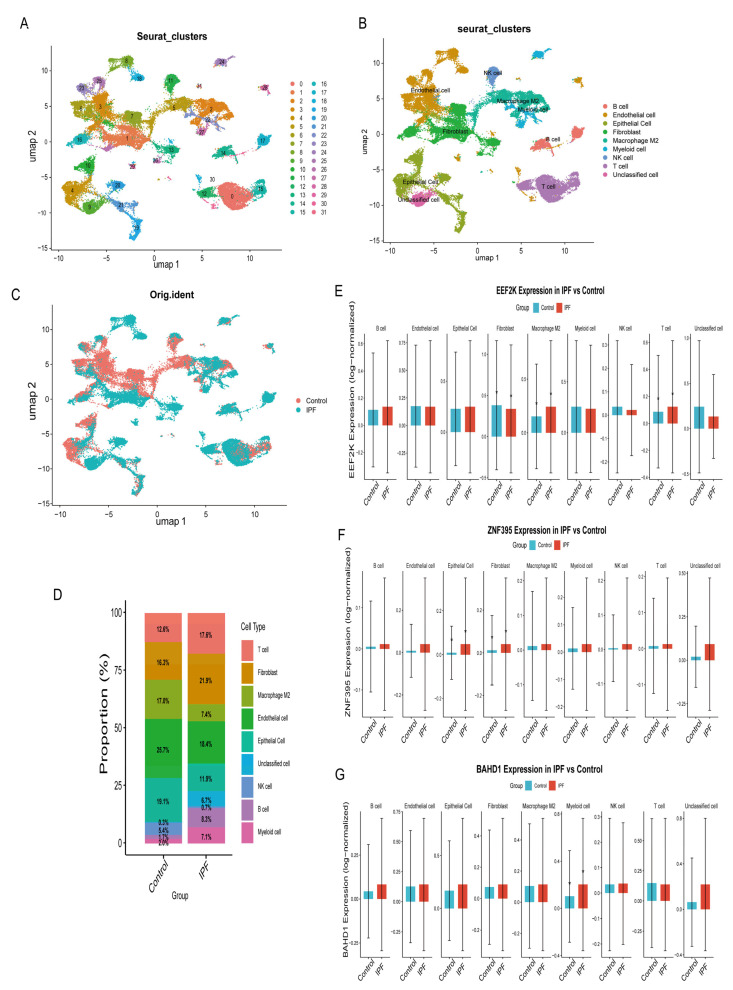
Single-cell transcriptomic profiling provides cellular context for core gene expression in IPF lungs. (**A**) UMAP plot showing the clustering of cells based on their gene expression profiles. Each dot represents a single cell, colored according to its assigned cell cluster. (**B**) UMAP plot displaying the distribution of annotated cell types across clusters. (**C**) UMAP plots showing single-cell transcriptomes from the IPF and control groups. (**D**) Proportional distributions of major cell populations in IPF and control samples. (**E**) Boxplots showing the cell-type–associated expression patterns of EEF2K in IPF and control samples. (**F**) Boxplots showing the cell-type–associated expression patterns of ZNF395 in IPF and control samples. (**G**) Boxplots showing the cell-type–associated expression patterns of BAHD1 in IPF and control samples. These single-cell analyses are intended to provide descriptive cellular localization and contextual expression patterns of the identified genes, rather than to imply statistically robust differential expression, functional specificity, or causal roles in particular cell types.

To systematically investigate IPF cellular alterations and core gene expression patterns, we obtained single-cell RNA-sequencing data (GSE283885, n = 15) from the GEO database and analyzed them using the Seurat package in R. After preprocessing and clustering, 31 cell clusters were identified and subsequently grouped into eight major annotated cell types plus an unclassified population (Figure 9A,B and Figures S3–S5). Comparative analysis of cell-type composition revealed marked differences between IPF and control lung samples (Figure 9C,D). IPF lungs exhibited increased proportions of T cells, fibroblasts, myeloid cells, and B cells, while reduced proportions of endothelial cells and epithelial cells were observed, consistent with previously reported cellular remodeling in fibrotic lung disease.

To explore the cellular context of the shared transcriptional features, we examined the expression patterns of ZNF395, EEF2K, and BAHD1 across major cell types. BAHD1 showed increased expression in myeloid cells in IPF lungs (*p* < 0.05) (Figure 9G). ZNF395 showed relative enrichment in epithelial cells and fibroblasts in IPF samples (*p* < 0.05) (Figure 9F). EEF2K displayed cell-type-dependent expression patterns, with increased expression in T cells and M2 macrophages and reduced expression in fibroblasts (*p* < 0.05) (Figure 9E).

Importantly, these observations reflect associations between gene expression and cell-type composition in IPF lungs, rather than direct evidence of cell-type-specific functional roles. The single-cell analysis is therefore intended to provide contextual support for the cellular localization of shared transcriptional signatures, rather than mechanistic or causal validation of cell-type-specific functions.

## 3. Discussion

Idiopathic pulmonary fibrosis (IPF) has increasingly been linked to systemic autoimmune and inflammatory conditions, including inflammatory bowel disease (IBD) [18]. These disease contexts may contribute to alveolar epithelial injury, inflammatory cytokine activation, and immune-cell recruitment to the lung. Among IBD subtypes, Crohn’s disease (CD) appears to show a closer association with pulmonary involvement than ulcerative colitis [11]. However, the molecular basis of this association remains poorly defined, and pulmonary extraintestinal manifestations may still be underrecognized in clinical practice [19].

In this study, we performed an integrative transcriptomic analysis across CD and IPF datasets and identified ZNF395, EEF2K, and BAHD1 as shared transcriptional features. These genes should not be interpreted as definitive diagnostic biomarkers for a clinically established CD-associated IPF entity. Instead, our analyses were designed to identify convergent immune–fibrotic transcriptional programs across two distinct disease contexts. Accordingly, the present findings should be viewed as hypothesis-generating and as a basis for future mechanistic and translational studies.

### 3.1. EEF2K and Immunometabolic Regulation

EEF2K is a Ca^2+^/calmodulin-dependent kinase that regulates protein translation by phosphorylating eEF2 at Thr56 [20]. This modification inhibits translation elongation and alters global protein synthesis. Beyond translational control, previous studies have implicated EEF2K in immunometabolic regulation, including macrophage polarization, T-cell function, and inflammatory responses [21]. In autoimmune and inflammatory settings, including rheumatoid arthritis, multiple sclerosis, and ulcerative colitis, EEF2K insufficiency has been associated with heightened immune activation through transcriptional and metabolic reprogramming of CD4^+^ T cells, CD8^+^ T cells, and macrophages [21]. Mechanistically, loss of EEF2K in CD4^+^ T cells impairs survival and proliferation while promoting IL-17 overproduction, whereas in CD8^+^ T cells it enhances glycolytic reprogramming through the Akt–mTOR–S6K pathway and augments effector function [22].

In fibrotic contexts, experimental inhibition of EEF2K has also been reported to exacerbate fibroblast-to-myofibroblast differentiation and myofibroblast proliferation [23]. In the present study, our data do not establish a direct causal role for EEF2K in fibroblast activation within the CD–IPF axis; rather, they provide reproducible evidence that EEF2K is associated with immune-related and fibrotic transcriptional states. Specifically, EEF2K expression showed consistent associations with T-cell populations in IPF lungs (Figure 7G,H and Figure 9E), alongside altered macrophage-related features (Figure 9D), and demonstrated reproducible within-disease discriminatory performance across independent CD and IPF cohorts (Figure 5B,F,G). Together, these findings support EEF2K as a candidate transcriptional feature of immune–fibrotic regulation, while its direct mechanistic contribution to fibroblast activation and cross-organ disease coupling remains to be determined.

### 3.2. ZNF395 and Immune-Responsive Transcription

ZNF395, a nuclear-cytoplasmic shuttling C2H2 zinc finger transcription factor, regulates downstream targets including chemokines CXCL10 and CXCL11 [24,25], and is critical for the induction of interferon-stimulated genes (ISGs) [26].

Although ZNF395 was overall downregulated at the bulk tissue level in human datasets, and its mouse ortholog was similarly reduced in the experimental fibrosis-associated models, single-cell analysis suggested relative enrichment of ZNF395 in epithelial and fibroblast compartments within IPF lungs. This apparent divergence may reflect different biological layers of transcriptional regulation: bulk downregulation may indicate a global tissue-level loss of homeostatic transcriptional programs during fibrotic remodeling, whereas relative compartmental enrichment in specific cell populations may suggest selective retention or compensatory activation of ZNF395-associated programs within remodeling niches. In this context, ZNF395 expression was relatively enriched in epithelial cells and fibroblasts (Figure 9F), suggesting a role in immune-responsive transcriptional adaptation within structurally remodeling tissue compartments rather than direct evidence of profibrotic activity. Beyond innate immunity, ZNF395 participates in adaptive immune regulation during viral infections, showing dynamic transcriptional changes in CD4^+^ and CD8^+^ T cells, which correlate with antiviral defense programs [27,28]. Our data indicated significant associations between ZNF395 expression and T-cell infiltration in IPF lungs; however, single-cell RNA sequencing did not reveal differential ZNF395 expression within T-cell subsets, suggesting that ZNF395 may serve as an indirect marker of immune cell transcriptional states rather than a direct effector. These observations support the concept that ZNF395 coordinates immune-responsive transcriptional activation, linking interferon signaling, chemokine-mediated recruitment, and T-cell activity in a context-dependent manner.

### 3.3. BAHD1 and Epigenetic Regulation

BAHD1 is a nuclear silencing factor containing a BAH domain that mediates heterochromatin formation and gene repression [29]. Functionally, BAHD1 contributes to chromatin organization and transcriptional constraint of immune-related loci [30]. Experimental knockdown of BAHD1 in HEK293 cells alters immune signaling pathways, including AP-1, NF-κB, and MAPK p38, indicating a potential regulatory role in immune-mediated transcription. In IPF, BAHD1 expression is associated with CD4^+^ and CD8^+^ T-cell populations (Figure 7E,F), and single-cell RNA sequencing identified differential expression in myeloid compartments (Figure 9G), supporting a link between BAHD1-mediated chromatin remodeling and immune transcriptional alterations. While BAHD1’s role may be primarily epigenetic rather than directly immunomodulatory, it may influence the overall immune-fibrotic landscape by constraining inflammatory gene programs and maintaining transcriptional homeostasis.

### 3.4. Potential Convergent Regulation of EEF2K, ZNF395, and BAHD1

Although EEF2K, ZNF395, and BAHD1 possess distinct molecular functions, integrative analyses suggest potential convergent or cooperative regulation. Correlation analysis of 28 shared gene signatures revealed coordinated expression patterns across CD and IPF datasets, implying embedding within interconnected immune- and stress-responsive networks. Functionally, EEF2K modulates immunometabolic and translational adaptation, ZNF395 orchestrates immune-responsive transcriptional activation, and BAHD1 imposes epigenetic constraints on immune-related loci. Together, these complementary layers of regulation suggest a conceptual framework wherein translational control, immune transcriptional programming, and chromatin-mediated repression jointly shape immune-inflammatory and fibrotic transcriptional states.

Immune infiltration and single-cell RNA sequencing further support overlapping cell population associations, including T cells and myeloid cells, suggesting coordinated regulation at the cellular level. However, direct molecular interactions or hierarchical regulatory relationships among these genes remain unproven. These findings underscore the importance of future mechanistic studies integrating functional perturbations, epigenomic profiling, and cell type-specific models to elucidate whether these factors interact directly or indirectly through shared pathways, chromatin landscapes, or metabolic circuits, and how such interactions may influence immune dysregulation and fibrotic remodeling in CD and IPF.

### 3.5. Limitations and Future Perspectives

Importantly, the present study does not establish diagnostic or predictive biomarkers for pulmonary fibrosis in patients with Crohn’s disease, as transcriptomic data from clinically confirmed CD–IPF comorbid cohorts are currently unavailable. Therefore, the findings should be regarded as hypothesis-generating rather than clinically actionable.

Several additional limitations should also be considered. First, all analyses were based on publicly available transcriptomic datasets, which may limit the generalizability of the results. In addition, the retrospective nature of these datasets warrants cautious interpretation until prospective studies and clinically well-annotated cohorts become available.

Second, the differential expression threshold used at the discovery stage was intentionally permissive in order to maximize sensitivity for identifying shared signals across distinct disease contexts. Although this strategy may introduce background noise, the final prioritized genes were subsequently refined through multiple complementary steps, including WGCNA-based module intersection, machine-learning–based feature prioritization, external cohort validation, and experimental validation, which together enhance the robustness of the final gene set.

Third, the cross-disease integrative strategy was performed primarily at the bulk tissue level. While this approach is appropriate for identifying broad shared transcriptional programs between CD and IPF, it does not resolve whether the observed molecular convergence arises from the same cellular populations across tissues. Consistent with this limitation, the single-cell RNA-seq analysis in the present study was exploratory and was intended to provide supportive cellular context rather than definitive cell-type-specific functional evidence. Future studies incorporating well-matched single-cell datasets from both diseases will be necessary to define shared cell-type-specific transcriptional signatures more precisely. In particular, identifying genes specialized in cell populations shared by both disease contexts may help further clarify the cellular basis of immune–fibrotic convergence.

Finally, although ZNF395, EEF2K, and BAHD1 were identified as shared transcriptional features associated with immune-related and fibrotic signatures, their precise functional roles in immune–fibrotic crosstalk remain to be established. The in vitro fibroblast experiments were included to assess fibrosis-associated expression changes in a tractable remodeling context, as fibroblasts represent a readily accessible cellular system for modeling fibrotic activation. However, this approach does not directly address the immune-cell-specific functions of these genes and should not be interpreted as functional validation in immune cells. Likewise, the in vivo model used in this study was based on a repetitive intratracheal bleomycin challenge, which likely reflects recurrent injury and fibrosis-associated remodeling rather than a canonical stable model of human IPF. Accordingly, the in vivo and in vitro findings should be interpreted as supportive validation of expression trends during fibrosis-associated remodeling, rather than as definitive functional evidence that these genes exert direct antifibrotic effects. Future mechanistic studies using immune cells, co-culture systems, cell-type-specific perturbation approaches, and better clinically defined cohorts will be required to clarify how these genes contribute to shared immune–fibrotic transcriptional programs across CD and IPF.

Future investigations should prioritize the establishment of well-phenotyped clinical cohorts, including Crohn’s disease patients with pulmonary involvement, to enable definitive validation of shared molecular signatures. In parallel, experimental studies dissecting downstream signaling pathways and cell-type–specific functions of ZNF395, EEF2K, and BAHD1 may provide deeper insight into immune–fibrotic transcriptional regulation and its potential translational relevance.

## 4. Materials and Methods

### 4.1. Data Sources and Processing

We analyzed nine publicly available datasets retrieved from the Gene Expression Omnibus (GEO) database (https://www.ncbi.nlm.nih.gov/geo (accessed on 7 January 2024)) to support a multi-level integrative analysis. The characteristics of the included datasets are summarized in Table S1. The main discovery and validation analyses in this study were based on bulk transcriptomic datasets, whereas single-cell RNA sequencing (scRNA-seq) was used only in a separate supportive analysis. For discovery analyses, three idiopathic pulmonary fibrosis (IPF) lung tissue datasets (GSE110147, GSE38958, and GSE24206) and two Crohn’s disease (CD) intestinal tissue datasets (GSE75214 and GSE94648) were included to identify shared transcriptional alterations between the two diseases. All analyses were performed in R (version 4.4.1), unless otherwise specified.

Raw expression data from these datasets were subjected to standard preprocessing procedures, including data quality evaluation, background correction, robust multi-array average (RMA) normalization, and gene annotation. To minimize technical variability across platforms and cohorts, batch effects among the three IPF datasets and two CD datasets were corrected using the ComBat algorithm implemented in the sva package [31] in R. After batch correction, integrated and standardized expression matrices were generated for downstream analyses. The effectiveness of batch correction was confirmed by improved clustering of samples according to biological condition rather than dataset origin (Figures S1 and S2). The combined IPF cohort comprised 109 patients and 62 control samples, whereas the CD cohort included 100 patients and 44 controls.

Independent datasets were subsequently incorporated for validation and complementary analyses, including peripheral blood mononuclear cells (PBMCs) from IPF patients (GSE28042), an independent IPF lung tissue dataset (GSE53845), and a CD colon tissue dataset (GSE36807). In addition, single-cell RNA-sequencing data from IPF lung samples (GSE283885) were analyzed to characterize cell-type–specific expression patterns of key genes identified in the bulk transcriptomic analyses. Detailed characteristics of all datasets, including sample size, tissue origin, and platform information, are summarized in Table S1.

### 4.2. Comparative Transcriptome Analysis

Differentially expressed genes (DEGs) were identified using the limma package in R [32] based on comparisons between disease and control samples within each IPF and CD dataset. To capture coordinated transcriptional patterns across diverse datasets, candidate genes were identified at the discovery stage using a lenient threshold (|log_2_FC| ≥ 0.25, adjusted *p* < 0.05). For downstream analyses, we prioritized genes showing consistent differential expression trends across the integrated IPF and CD cohorts. DEG results were visualized using volcano plots and heatmaps generated using ggplot2 and pheatmap [33], and gene overlaps were illustrated using Venn diagrams.

### 4.3. Weighted Gene Co-Expression Network Analysis (WGCNA)

Weighted gene co-expression network analysis (WGCNA) was performed using the WGCNA package in R to identify disease-associated gene co-expression modules in idiopathic pulmonary fibrosis (IPF) and Crohn’s disease (CD). Prior to network construction, genes with low expression variability were filtered based on median absolute deviation (MAD > 0), and sample quality was evaluated using the goodSamplesGenes function.

Scale-free co-expression networks were constructed separately for the IPF and CD datasets, following standard WGCNA procedures [34]. Soft-thresholding powers were determined independently for each dataset based on scale-free topology fit and mean connectivity criteria using the pickSoftThreshold, resulting in β = 18 for the integrated IPF dataset and β = 7 for the combined CD cohort (Figure 3A,B).

Genes were subsequently hierarchically clustered into modules using dynamic tree cutting. Each module was summarized by its module eigengene (ME), and associations between MEs and disease status were assessed using Spearman correlation analysis. Modules showing the strongest associations with disease status were selected for downstream analyses. Because the primary aim of this study was to identify shared disease-associated genes across IPF and CD, rather than topological hub genes within a single disease-specific network, we did not initially restrict candidate selection to classical intramodular hub genes defined solely by within-module connectivity. Instead, genes from the disease-relevant modules were retained for cross-disease intersection analysis, followed by machine-learning–based prioritization. Module membership (MM) and gene significance (GS) values were calculated to further prioritize disease-associated genes within key modules.

### 4.4. Functional Enrichment Analysis

Gene Ontology (GO) and Kyoto Encyclopedia of Genes and Genomes (KEGG) pathway enrichment analyses were conducted using the clusterProfiler package in R [33,35] to characterize the biological functions of shared differentially expressed genes (DEGs). All genes included in the differential expression analysis were used as the background gene set. Multiple testing correction was performed using the Benjamini–Hochberg method, and enrichment terms with an adjusted *p* value < 0.05 were considered statistically significant.

### 4.5. Gene Set Enrichment Analysis (GSEA)

Gene set enrichment analysis (GSEA) was performed to investigate coordinated pathway-level expression patterns associated with the identified core genes. GSEA was implemented using the clusterProfiler package in R, with curated gene sets obtained from the Molecular Signatures Database (MSigDB). Genes were ranked according to their log_2_ fold-change values derived from differential expression analysis.

Enriched pathways were considered statistically significant based on the following criteria: absolute normalized enrichment score (|NES|) > 1, adjusted *p* value < 0.05, and false discovery rate (FDR) ≤ 0.25.

### 4.6. Machine Learning Analysis

To prioritize robust shared transcriptional features across Crohn’s disease (CD) and idiopathic pulmonary fibrosis (IPF), three complementary machine learning algorithms, random forest (RF) [36], least absolute shrinkage and selection operator (LASSO) regression [37], and extreme gradient boosting (XGBoost) [38], were applied as feature selection and ranking tools. All machine learning analyses were performed in R using the randomForest, glmnet, and xgboost packages.

The 28 shared disease-associated module genes identified from the cross-disease WGCNA were used as the common candidate pool. Feature selection was performed separately in the integrated IPF cohort and the combined CD cohort using RF, LASSO, and XGBoost. Genes consistently prioritized across algorithms within each disease context and overlapping across the two disease cohorts were defined as machine-learning–prioritized genes.

These algorithms were used to identify and prioritize stable disease-associated genes rather than to construct disease-prediction models. RF and XGBoost were employed to rank variable importance and select the top 15 candidate features, while LASSO regression was applied for penalized dimensionality reduction and feature selection [39,40]. Receiver operating characteristic (ROC) curves were generated using the pROC package in R [39] to assess the consistency of within-disease discrimination across independent cohorts. These performance metrics were used solely to evaluate feature robustness and reproducibility, rather than to infer clinical diagnostic utility or cross-disease predictive capability.

### 4.7. Nomogram Model and Validation

A nomogram integrating the five machine learning–prioritized genes was constructed using the rms package in R [40] as a visual and integrative framework to summarize the relative contributions of the identified shared genes. Model performance was evaluated using receiver operating characteristic (ROC) curves, calibration plots, clinical impact curves (CICs), and decision curve analysis (DCA) [39,41,42], with these metrics interpreted in an exploratory and descriptive manner.

External validation was performed using independent IPF (GSE53845) and CD (GSE36807) cohorts to assess within-disease consistency. In addition, cross-disease evaluation using a peripheral blood mononuclear cell (PBMC) dataset (GSE28042) was conducted to examine the robustness and generalizability of the shared transcriptional signature across distinct disease and tissue contexts, rather than to imply direct clinical predictive applicability.

### 4.8. Immune Infiltration Analysis

Immune cell composition in IPF transcriptomes was estimated using CIBERSORT in R [43], based on the LM22 leukocyte gene signature matrix. Because the CIBERSORT LM22 reference matrix is optimized for leukocyte deconvolution in bulk tissue and the integrated CD datasets exhibited marked tissue heterogeneity related to intestinal mucosal composition, immune deconvolution was conservatively restricted to the IPF lung tissue cohort. This analysis was applied as an exploratory deconvolution approach to infer relative immune cell proportions within bulk lung transcriptomic data.

Differences in the estimated proportions of 22 immune cell subsets between IPF patients and controls were assessed using the Wilcoxon rank-sum test, with *p* < 0.05 considered statistically significant. Correlations among immune cell subsets were visualized using the corrplot package, and associations between core gene expression and immune cell infiltration were evaluated using Spearman correlation analysis. These analyses were performed to characterize immune–transcriptional relationships in a descriptive and hypothesis-generating manner, rather than to infer causal interactions.

### 4.9. Single-Cell RNA-Seq Analysis

We obtained single-cell RNA-sequencing data from IPF and control lung tissues (GSE283885) and analyzed them using the Seurat package in R [44]. The dataset comprised 15 lung tissue samples, including 9 samples from patients with idiopathic pulmonary fibrosis at the time of lung transplantation and 6 samples from healthy donor controls. Cell counts before and after quality filtration for each sample are summarized in Table S2.

Quality control was performed to exclude low-quality cells and potential doublets. Cells expressing fewer than 200 genes or more than 5000 genes were removed, and cells with mitochondrial gene content exceeding 5% were excluded. In addition, genes were required to be expressed in at least three cells to be retained for downstream analysis. Quality control metrics, including the number of detected genes, total counts, and mitochondrial gene percentage, were visualized using VlnPlot() and FeatureScatter() functions.

Following quality control, gene expression data were normalized using the LogNormalize method with a scaling factor of 10,000. Highly variable genes were identified using the variance-stabilizing transformation (vst) method, and the top 3000 variable genes were selected for downstream analyses. These genes were scaled using ScaleData(), and principal component analysis (PCA) was performed using RunPCA() based on the 3000 highly variable genes.

The number of principal components used for downstream analyses was determined by inspection of the elbow plot, and the first 49 principal components were retained. A shared nearest neighbor (SNN) graph was constructed using FindNeighbors(), followed by graph-based clustering using FindClusters() with a resolution parameter of 0.5. Uniform manifold approximation and projection (UMAP) was applied for nonlinear dimensionality reduction and visualization of cell clusters.

Cluster-specific marker genes were identified using the FindAllMarkers() function with the parameters only.pos = TRUE, min.pct = 0.25, and logfc.threshold = 0.25. Cell type annotation was performed based on established lung cell markers: T cells (CD2, CD3D); B cells (MZB1, CD79A); NK cells (FCGR3A); myeloid cells (CD68, LYZ, ITGAX); fibroblasts (DCN, TAGLN, COL1A1); endothelial cells (CDH5, KDR); epithelial cells (EPCAM, KRT5); and macrophage subtypes, including M1-like macrophages (CD80, CD86) and M2-like macrophages (CD163, CD206). Clusters that did not robustly express canonical markers of known lung cell types were conservatively annotated as unclassified cells and were excluded from downstream biological interpretation.

Notably, epithelial cells were annotated as a broad epithelial compartment rather than being subdivided into alveolar type I (AT1) and type II (AT2) cells, due to the limited resolution of canonical AT1/AT2 marker genes in this dataset and to avoid overinterpretation of cell subtype identities.

### 4.10. Cell Culture and In Vitro Assays

Mouse L929 fibroblasts were obtained from the American Type Culture Collection (ATCC, Manassas, VA, USA) and maintained in Dulbecco’s modified Eagle medium (DMEM) supplemented with 10% FBS under standard culture conditions (37 °C, 5% CO_2_). The medium was replaced every 2 to 3 days to ensure nutrient availability. Upon reaching 80% confluency, the cells were dissociated with 0.25% trypsin-EDTA and subcultured at a 1:3 split ratio. To maintain phenotypic stability, the cells were used within 30 passages after thawing. We treated murine L929 fibroblasts with TGF-β1 (10 ng/mL; MCE, Monmouth Junction, NJ, USA) for 24 h and performed qPCR and Western blotting to detect the mRNA and protein expression of Col1a1, Acta2, Eef2k, Bahd1, and zfp395.

### 4.11. Animal Model

Eight healthy male C57BL/6 mice (6–8 weeks old) were randomly divided into control (C) and model (M) groups (n = 4 per group). A repetitive bleomycin challenge model was established by intratracheal administration of bleomycin (1 mg/kg) on days 0, 7, 14, and 21, while control mice received saline. On day 28, lung tissues were collected for HE/Masson staining; hydroxyproline assay; and qRT-PCR/Western blot analyses of Col1a1, Acta2, TGF-β1, Eef2k, Bahd1, and zfp395 expression; IHC analyses of Eef2k, Bahd1, and zfp395; and IF analyses of α-SMA, CD4 and CD8.

### 4.12. qRT–PCR and Western Blot

The amounts of Col1a1, Acta2, Eef2k, Bahd1, and zfp395 were determined via qRT–PCR and Western blotting. Total RNA was extracted from lung tissues or Mouse L929 fibroblasts via TRIzol reagent (Invitrogen, Carlsbad, CA, USA) following the manufacturer’s protocol. cDNA synthesis was subsequently performed via the PrimeScript™ RT Reagent Kit (Servicebio, Wuhan, HB, China). Quantitative reverse transcription PCR (qRT–PCR) was conducted using 2 × Q3 SYBR qPCR Master Mix (TOLOBIO, Shanghai, China). The relative mRNA expression levels were normalized to those of GAPDH, which was used as the endogenous control. The sequences of primers used in this study are listed in Table 1.

After total protein was extracted from lung tissue or L929 fibroblasts, the protein concentration was determined via a BCA Protein Assay Kit (Beyotime, Biotechnology, Shanghai, China). Thirty micrograms of total protein from each sample were separated by 10% SDS–PAGE and transferred onto PVDF membranes. The membranes were then blocked for 2 h and incubated overnight at 4 °C with primary antibodies against Col1a1 (1:1000, UpingBio, Hangzhou, Zhejiang, China), α-SMA (1:1000, UpingBio, Hangzhou, Zhejiang, China), TGF-β1 (1:1000, UpingBio, Hangzhou, Zhejiang, China), E-cadherin (1:2000, Abcam, Cambridge, UK), eEF2K (1:1000, Proteintech, Chicago, IL, USA), Zfp395 (1:1000, Affinity, Biosciences, Jiangsu, China), BAHD1 (1:1000, HUABIO, Hangzhou, Zhejiang, China), or GAPDH (1:5000, ABclonal, Wuhan, Hubei, China). E-cadherin was measured as an additional epithelial-associated marker but was not interpreted as a canonical fibrotic marker in the primary analyses. The membranes were subsequently incubated with HRP-conjugated secondary antibodies (1:5000, Elabscience, Wuhan, Hubei, China) for 2 h. The protein bands were visualized via a ChemiDoc Touch Imaging System (Bio-Rad, Laboratories, Hercules, CA, USA) and quantified via ImageJ software (version 1.53a).

### 4.13. Pathological and Biochemical Assays

For the analysis of the mouse pulmonary fibrosis model, 4% paraformaldehyde-fixed paraffin-embedded left lung lobe tissue was used for pathological examination, hematoxylin–eosin (HE) staining, and Masson’s trichrome staining.

The hydroxyproline content in mouse lung tissues was quantified via a hydroxyproline assay kit (E-BC-K062-M, Elabscience, Wuhan, Hubei, China). Briefly, the lung tissues were hydrolyzed in 6 M HCl at 120 °C for 3 h. The hydrolysates were then neutralized and reacted with chloramine-T and dimethylaminobenzaldehyde according to the manufacturer’s instructions. The absorbance was measured at 560 nm, and the hydroxyproline content was calculated against a standard curve and normalized to the total lung weight.

### 4.14. Immunohistochemistry and Immunofluorescence

Immunohistochemistry (IHC) was performed on consecutive 4-μm-thick paraffin-embedded sections of lung tissue mounted on poly-L-lysine-coated slides. The sections were deparaffinized in xylene and rehydrated through a graded ethanol series. Antigen retrieval was performed in a microwave oven using antigen retrieval buffer (pH 9.0). Endogenous peroxidase activity was blocked by incubating the sections with 3% hydrogen peroxide for 25 min at room temperature in the dark. After blocking with 3% bovine serum albumin (BSA) for 30 min at room temperature, the sections were incubated overnight (18–20 h) at 4 °C with primary antibodies against eEF2K (1:100) (13510-1-AP, Proteintech, Chicago, IL, USA), Zfp395 (1:50) (DF14677, Affinity, Biosciences, Jiangsu, China), and BAHD1 (1:100) (HA500589, HUABIO Hangzhou, Zhejiang, China). After washing, the sections were incubated with the corresponding HRP-conjugated secondary antibodies for 50 min at room temperature in the dark. DAB substrate (C1001, BaiQianDu Biotechnology, Wuhan, Hubei, China) was used for color development under microscopic observation. The sections were then rinsed with water, counterstained with hematoxylin (C2101, BaiQianDu Biotechnology, Wuhan, Hubei, China), differentiated in 1% hydrochloric alcohol, blued in ammonia water, washed under running water, dehydrated through graded ethanol, air-dried, and mounted with neutral gum.

### 4.15. Statistical Analysis

The data were analyzed with GraphPad Prism v8.0.2. The results are shown as the means ± SDs. Between-group differences were assessed by two-tailed unpaired *t*-tests. *p* < 0.05 was considered significant.

## 5. Conclusions

In summary, integrative transcriptomic analyses and machine learning–based feature selection identified ZNF395, EEF2K, and BAHD1 as reproducibly shared genes across CD and IPF. These genes were associated with immune–fibrotic transcriptional programs across independent disease contexts and were supported by single-cell expression patterns and fibrosis-associated models. However, the present study does not establish them as disease-specific or predictive biomarkers for pulmonary fibrosis in patients with CD. However, the present study does not establish these genes as disease-specific or predictive biomarkers for pulmonary fibrosis in patients with CD. Instead, our findings support shared transcriptional logic across intestinal inflammation and pulmonary fibrosis, rather than direct patient-level pathogenic continuity between Crohn’s disease and idiopathic pulmonary fibrosis. These results provide a framework for future validation in clinically defined CD–IPF cohorts.

## Figures and Tables

**Table 1:** List of primers.

Primers	Forward (5′-3′)	Reverse (5′-3′)
Col1a1	TTCGTGACCGTGACCTTGAG	CGATCTCGTTGGATCCCTGG
Acta2	CCCAGACATCAGGGAGTAATGG	TCTATCGGATACTTCAGCGTCA
Tgfb-1	CTTCAATACGTCAGACATTCGGG	GTAACGCCAGGAATTGTTGCTA
Zfp395	TGAGCCCATCTTCTCTGTTTCC	CAATGAAAGAGCAGCAAGGGAC
Eef2k	TCACCGGGACTCTGAGAATAGT	TCGGGCCACTAAAATCATGGAA
Bahd1	CGGCCAATTGGAATTTCCTCTC	AAGTGCTCAGGCCTGTAATACC
Gapdh	CCTTCCGTGTTCCTACCC	GCCTGCTTCACCACCTTC

## Data Availability

This study utilized multiple datasets from the Gene Expression Omnibus (GEO), including the following accession numbers: GSE110147, GSE38958, GSE24206, GSE75214, GSE94648, GSE28042, GSE53845, GSE283885 and GSE36807. All the computational scripts used for data processing and analysis are available in the Supplementary Materials (Additional Table S1).

## Supplementary Materials

The following supporting information can be downloaded at: https://www.mdpi.com/article/10.3390/ijms27104428/s1.

## Abbreviations

CD = Crohn’s disease; EIMs = extraintestinal manifestations; IPF = idiopathic pulmonary fibrosis; WGCNA = weighted gene co-expression network analysis; DEGs = differentially expressed genes; GSEA = gene set enrichment analysis; DCA = decision curve analysis; CIC = clinical impact curve; UC = ulcerative colitis.
