# Supplementary material for: Integrative Transcriptomic Analysis Identifies Shared Immune–Fibrotic Transcriptional Programs Across Crohn’s Disease and Idiopathic Pulmonary Fibrosis
Source: Int J Mol Sci. 2026 May 15;27(10):4428. doi: 10.3390/ijms27104428 (PMC13207281; doi:10.3390/ijms27104428)
Supplement: Supplementary file 1 [file ijms-27-04428-s001.zip › ijms-4252510-supplementary.pdf]

Figure S1

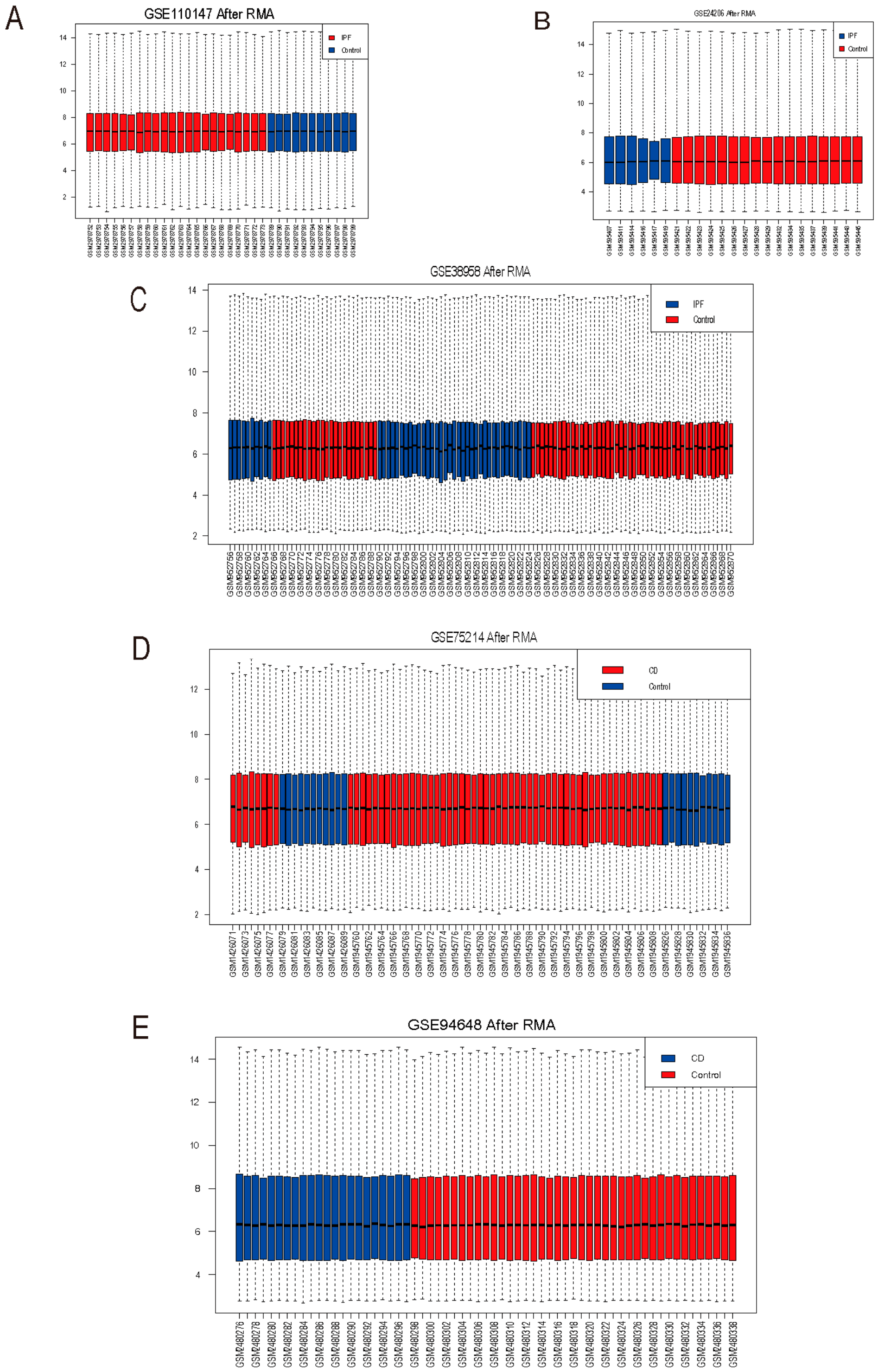

Figure S1. Boxplots of gene expression data after RMA normalization. (A) GSE110147 after RMA normalization, (B) GSE24206 after RMA normalization, (C) GSE38958 after RMA normalization, (D)GSE75214 after RMA normalization, (E) GSE94648 after RMA normalization

Figure S2

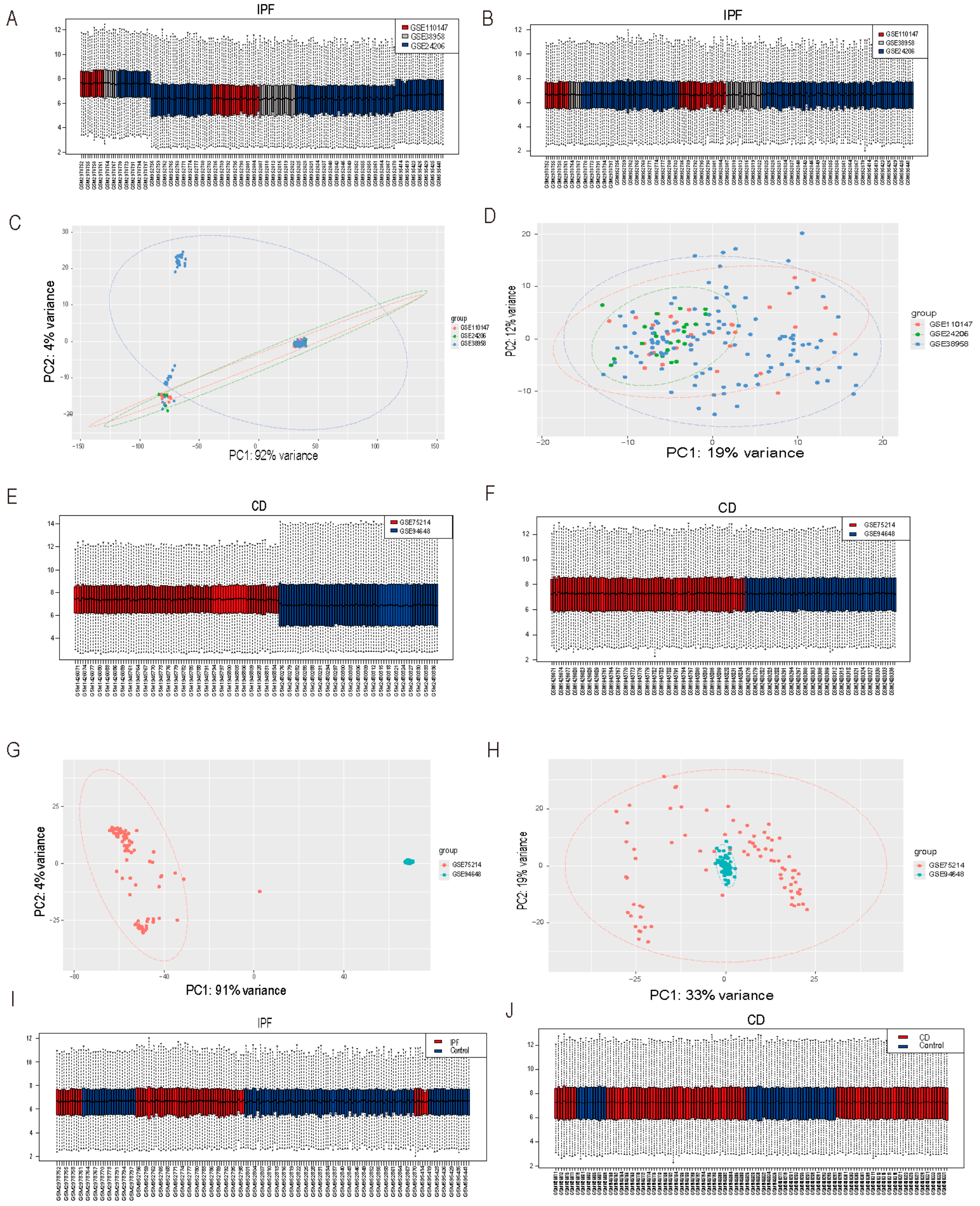

Figure S2. The integration of IPF datasets and CD datasets. (A) Boxplots of three raw IPF datasets before batch-effect correction. (B) Boxplots of three raw IPF datasets after batch-effect correction. (C) PCA of three raw IPF datasets before batch-effect correction. (D) PCA of three raw IPF datasets after batch-effect correction. (E) Boxplots of two raw CD datasets before batch-effect correction. (F) Boxplots of two raw CD datasets after batch-effect correction. (G) PCA of two raw CD datasets before batch-effect correction. (H) PCA of two raw CD datasets after batch-effect correction. (I) Annotation of IPF and corresponding control samples for all three IPF datasets. (J) Annotation of CD and corresponding control samples for both CD datasets.

Figure S3

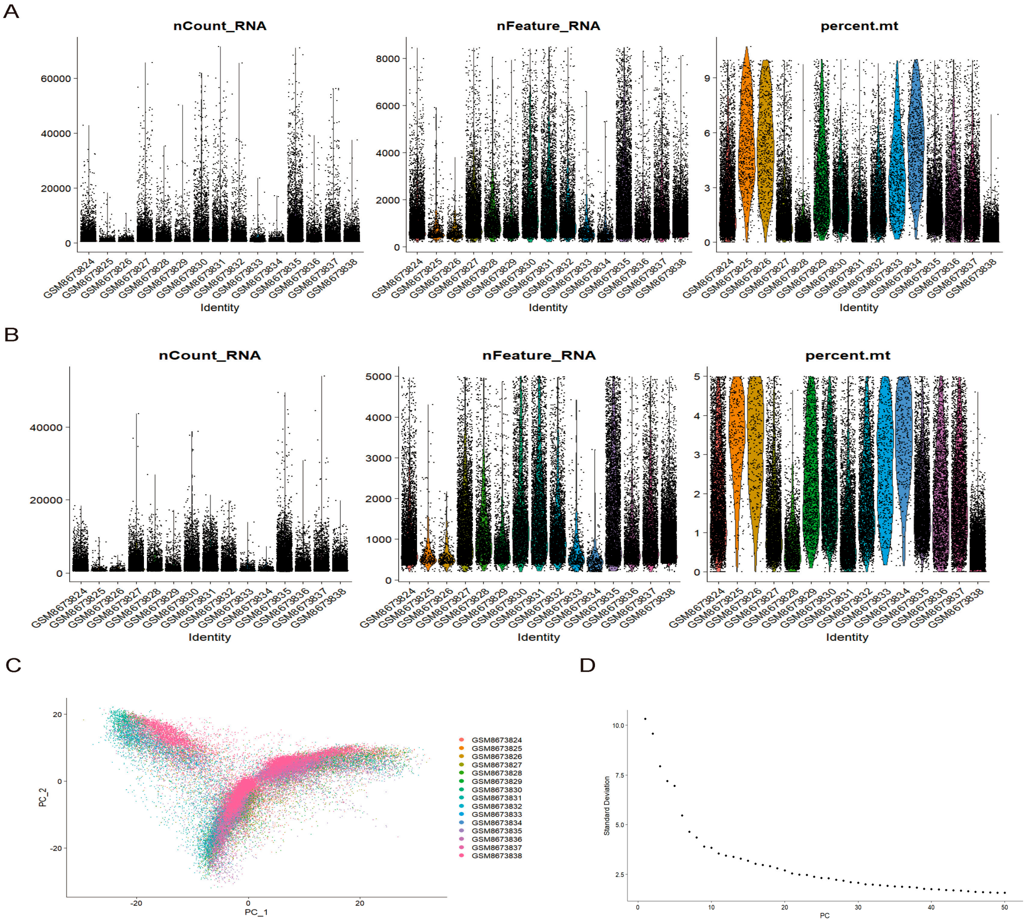

Figure S3. Quality control, normalization, feature selection, and principal component analysis (PCA) dimensionality reduction of single-cell RNA sequencing data. (A) The violin plot for quality control of single-cell RNA sequencing data before filtering. (B) The violin plot for quality control of single-cell RNA sequencing data after filtering. (C) PCA for single-cell RNA sequencing data after dimensionality reduction. (D) Variability explained by characteristic variables, with PCs on the x-axis and variance proportion on the y-axis.

Figure S4

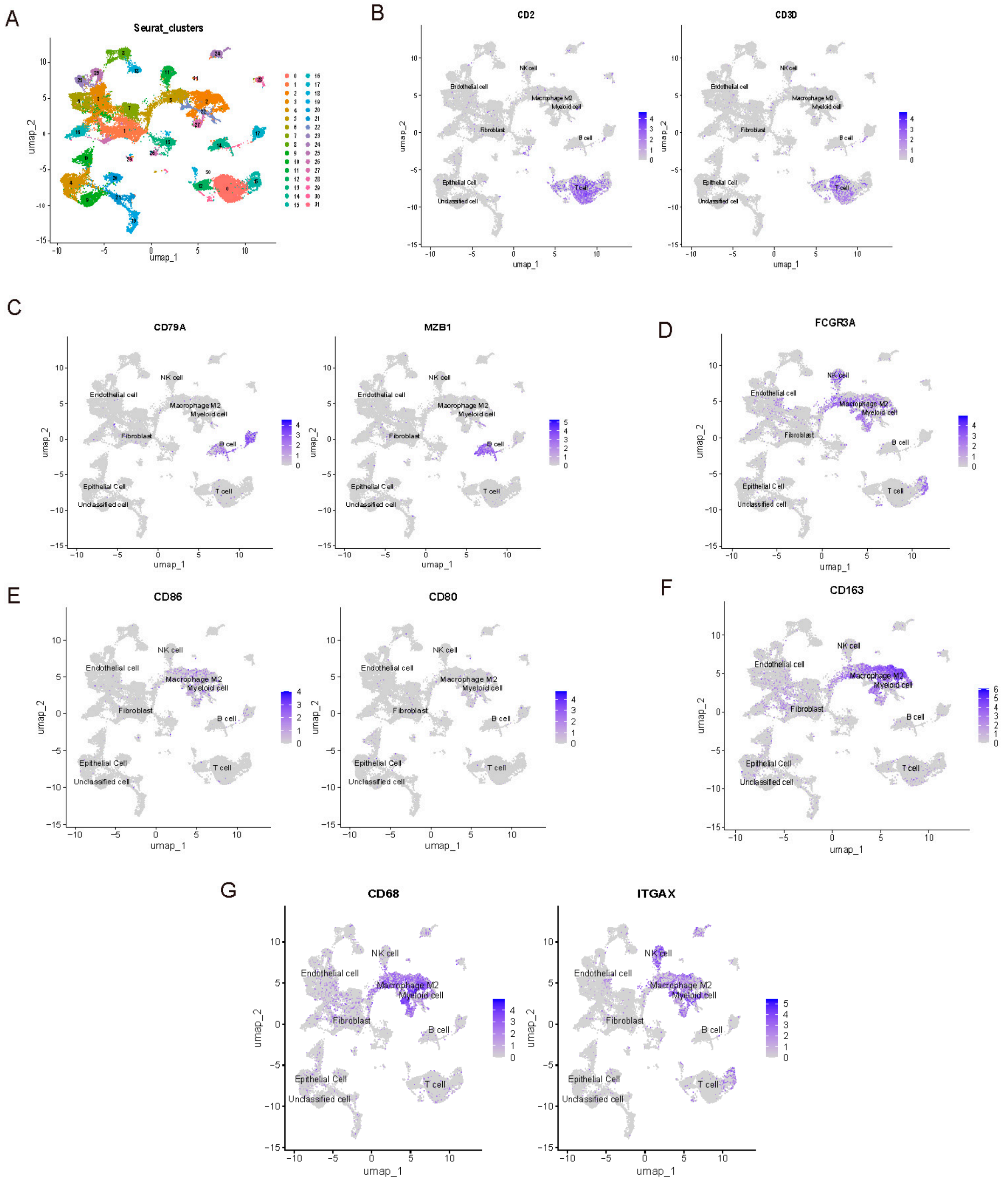

Figure S4. Cell cluster expression patterns and UMAP visualization of selected marker genes. (A) UMAP plot showing the clustering of cells based on their gene expression profiles. Each dot represents a single cell, colored by its assigned cluster. (B) UMAP plots showing the distribution of T cells across different dimensions, with color indicating the expression levels of T cells marker genes. (C) UMAP plots showing the distribution of B cells across different dimensions, with color indicating the expression levels of B cells marker genes. (D) UMAP plots showing the distribution of NK cells across different dimensions, with color indicating the expression levels of NK cells marker genes. (E) UMAP plots showing the distribution of M1 macrophages across different dimensions, with color indicating the expression levels of M1 macrophages marker genes. (F) UMAP plots showing the distribution of M2

macrophages across different dimensions, with color indicating the expression levels of M2 macrophages marker genes. (G) UMAP plots showing the distribution of Myeloid cells cells across different dimensions, with color indicating the expression levels of Myeloid cells marker genes.

Figure S5

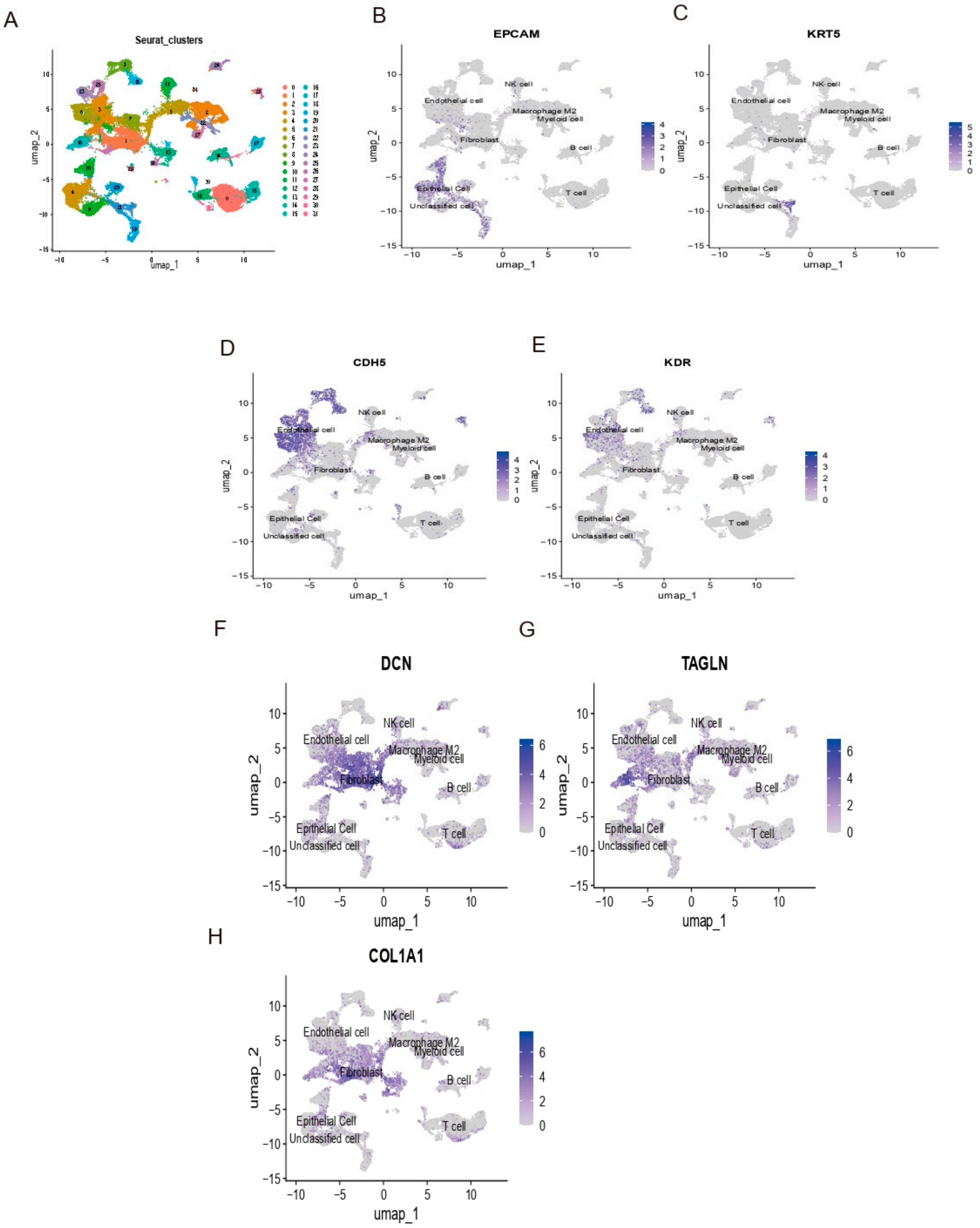

Figure S5. Cell cluster expression patterns and UMAP visualization of selected marker genes. (A) UMAP plot showing the clustering of cells based on their gene expression profiles. Each dot represents a single cell, colored by its assigned cluster. (B-C) UMAP plots showing the distribution of epithelial cells across different dimensions, with color indicating the expression levels of epithelial cells marker genes. (D-E) UMAP plots showing the distribution of endothelial cells across different dimensions, with color indicating the expression levels of endothelial cells marker genes. (F-H) UMAP plots showing the distribution of fibroblasts across different dimensions, with color indicating the expression levels of fibroblasts marker genes.

Table S1. Characteristics of the studies composing the nine-dataset gene expression compendium.

Dataset	Platform	Types of samples	Region	sample
GSE110147	Affymetrix Human Gene 1.0 ST Array	Human lung tissue	London, Canada	IPF：22<br>Control：11
GSE38958	Affymetrix Human Exon 1.0 ST Array	Human peripheral blood mononuclear cells (PBMCs)	Chicago, Illinois, USA	IPF：70<br>Control：45
GSE24206	Affymetrix Human Genome U133 Plus 2.0 Array	Human lung tissue	Durham, North Carolina, USA	IPF：17<br>Control：6
GSE75214	Affymetrix Human Gene 1.0 ST Array	Human colonic tissue	Hasselt, Belgium	CD：59<br>Control：22
GSE94648	Affymetrix Human Genome U133 Plus 2.0 Array	Human colonic tissue	Barcelona, Spain	CD：41<br>Control：22
GSE53845	Agilent-014850 Whole Human Genome Microarray 4x44K G4112F	Human lung tissue	South San Francisco, California, USA	IPF:40<br>Control:8
GSE36807	Affymetrix Human Genome U133 Plus 2.0 Array	Human colonic tissue	London, United Kingdom.	CD:13<br>Control:7
GSE28042	Agilent-014850 Whole Human Genome Microarray 4x44K G4112F	Human peripheral blood mononuclear cell (PBMC)	New Haven, USA	IPF:75<br>Control：19
GSE283885	Illumina NovaSeq 6000	Human lung tissue	Columbus, USA	IPF:6<br>Control:9

Table S2: Cell counts before and after filtration of the samples.

sample	Raw count	Clean count	Percentage (%)
GSE8673824	5438	4784	84.97
GSE8673825	618	279	45.15
GSE8673826	611	294	48.12
GSE8673827	4738	4371	92.25
GSE8673828	1900	1851	97.12
GSE8673829	2165	1600	73.90
GSE8673830	3951	3499	88.56
GSE8673831	4159	3846	92.47
GSE8673832	3520	3189	90.60
GSE8673833	729	518	71.06
GSE8673834	753	342	45.42
GSE8673835	5626	4895	87.01
GSE8673836	2766	2409	87.09
GSE8673837	4370	3915	89.59
GSE8673838	11369	11336	99.71
